# Geospatial dataset of oil wells and socio-environmental vulnerability indicators in coastal urban territories

**DOI:** 10.1016/j.dib.2026.112960

**Published:** 2026-06-11

**Authors:** David Freire, Paulo Escandón-Panchana, Jorge Magallanes-Tomalá, Andrés Velastegui-Montoya, Jenny Escandón-Panchana

**Affiliations:** a Faculty of Mechanical Engineering and Production Sciences, ESPOL Polytechnic University, Campus Gustavo Galindo, Km. 30.5 Vía Perimetral, Guayaquil, 090902, Ecuador; b Laboratory of Geoinformation and Remote Sensing, Faculty of Engineering in Earth Sciences, ESPOL Polytechnic University, Campus Gustavo Galindo, Km. 30.5 Vía Perimetral, Guayaquil, 090902, Ecuador; c Escuela de Ciencias Ambientales, Universidad Espíritu Santo, Samborondón, Ecuador; d Departamento de Ingeniería Cartográfica y Topografía, Universidad Politécnica de Madrid, Crta. Valencia, 28031, Madrid, Spain; e Facultad de Ciencias del Mar, Universidad Estatal Península de Santa Elena (UPSE), La Libertad, 240204, Ecuador; f Faculty of Engineering in Earth Sciences, ESPOL Polytechnic University, Campus Gustavo Galindo, Km. 30.5 Vía Perimetral, Guayaquil, 090902, Ecuador; g Independent Researcher, La Libertad, Santa Elena 240204, Ecuador

**Keywords:** Oil wells, Proximity, Environmental vulnerability, Geospatial, Coastal urban territories, Environmental risk assessment

## Abstract

Data on oil wells, including their identification, technical characteristics and environmental context, constitute an important input for the analysis of socio-environmental vulnerability in territories with a historical presence of hydrocarbon activity. In coastal urban contexts, population growth, urban and housing expansion, and land-use transformations have progressively increased the proximity of this infrastructure to urbanised areas, industrial zones associated with salt production, and public spaces such as roads, parks, and fishing ports. In Ecuador, the coastal canton of Salinas has approximately 228 wells drilled since the beginning of the 20th century, currently distributed in urban and rural sectors of the coastal profile. Despite their historical andterritorial relevance, geospatial datasets integrating information on oil wells with socio-environmental indicators are scarce in coastal urban territories. This study aims to develop a geospatial dataset that integrates the locations of oil wells with demographic, environmental, and urban infrastructure variables to facilitate vulnerability analysis in coastal urban territories. The information was compiled from local government technical reports and data sheets, subsequently cleaned and georeferenced using Geographic Information Systems (GIS) and GPS navigation systems to validate its position and territorial context. The dataset includes 11 standardised variables, including well identification, geological formation, extraction system, well status, safety distances from oil infrastructure (oil/multi-product pipeline, wellhead, and gas pipeline), population density, proximity to urban infrastructure and sensitive ecosystems, and interference from hydrocarbon transport lines. These variables were normalised and integrated to generate a spatial vulnerability index on an ordinal scale of 1 to 10, assigned to each of the 228 wells included in the dataset. The resulting weighting indicates that 68.42% of the wells present medium to high levels of socio-environmental vulnerability. The dataset is presented in tabular and geospatial formats, structured to facilitate its reuse in environmental risk studies, socio-spatial analysis, territorial planning and comparative evaluation in other local and regional coastal urban territories with similar characteristics.

Specifications Table

**Table:** 

Subject	Earth & Environmental Sciences
Specific subject area	Georeferencing and socio-environmental characterisation of 228 oil wells located in the Salinas canton, Santa Elena province, Ecuador. The dataset integrates technical variables, such as geological formation, extraction system, well operational status, and pipeline interference. It also includes spatial metrics of distance and proximity, such as safety distance and proximity to urban infrastructure, as well as social and environmental indicators, including population density and the presence of sensitive ecosystems.
Type of data	Comma-Separated Values file. GIS shape file
Data collection	The data were compiled from oil well technical data sheets, historical reports, and technical documentation provided by the Decentralised Autonomous Government (GAD, acronym in Spanish) of the Salinas canton. Subsequently, technical field visits were conducted to verify and update the wells' geographic coordinates and to validate the distances between safety zones, populated areas, and surrounding infrastructure.
Data source location	Institution: GAD of the Salinas canton City/Town/Region: Salinas, Santa Elena Province Country: Ecuador Salinas 2° 13′ S and 80°58′ W www.salinas.gob.ec
Data accessibility	Repository name: Mendeley Data Data identification number: 10.17632/bw2bgtjw98.1 Direct URL to data: https://data.mendeley.com/datasets/bw2bgtjw98/1 Instructions for accessing these data: The shapefile (DatasetOilWell.gpkg) is provided in the repository and can be loaded into a GIS program (QGIS).
Related research article	G. Herrera-Franco, P. Escandón-Panchana, F.J. Montalván, A. Velastegui-Montoya, CLUE-S model based on GIS applied to management strategies of territory with oil wells—Case study: Santa Elena, Ecuador, Geography and Sustainability 3 (2022) 366–378. https://doi.org/10.1016/j.geosus.2022.11.001. G. Herrera-Franco, F.J. Montalván, A. Velastegui-Montoya, J. Caicedo-Potosí, Vulnerability in a Populated Coastal Zone and Its Influence by Oil Wells in Santa Elena, Ecuador, Resources 11 (2022) 70. https://doi.org/10.3390/resources11080070.

## 1 Value of the Data

• This dataset provides detailed geospatial information on the locations and technical characteristics of oil wells in the Salinas canton, enabling assessment of their proximity to and spatial interactions with homes, urban infrastructure, and sensitive ecosystems. Its structure facilitates the identification of areas where hydrocarbon infrastructure interacts with populated zones, providing relevant technical input for land-use planning and environmental risk management.
• The integration of physical, social, and environmental variables allows for the analysis of vulnerability associated with hydrocarbon extraction from a spatial and integrated perspective. The dataset can be used in risk assessment models, population exposure studies, potential pollution analysis, and the design of environmental mitigation measures.
• The information is also useful for land-use planning, tourism management in coastal environments, and the conservation of ecosystems and areas of geological value. It also facilitates the identification of areas with greater environmental sensitivity, which may require ongoing monitoring, specific regulations, or differentiated land-use management strategies.
• Finally, the standardised and georeferenced format of the dataset facilitates its reuse in comparative analyses between wells, spatial simulations, and multi-criteria evaluations. This approach broadens its applicability in future research, particularly for evidence-based decision-making processes in coastal urban areas with a history of petroleum infrastructure.

## 2 Background

Oil and gas exploitation has played a significant role in economic growth and in the development of energy and industrial infrastructure in several territories. However, associated activities such as site preparation, drilling, production, and well maintenance can affect soil, water, and surrounding ecosystems, as well as pose risks to human health and environmental quality [1,2]. These concerns intensify when hydrocarbon infrastructure is located in urban or peri-urban environments, where proximity to housing, critical infrastructure, and basic services increases the population's socio-environmental exposure [3].

In response to this problem, during the last decades, spatial analysis approaches based on Geographic Information Systems (GIS) and proximity metrics have been developed and consolidated to assess patterns of exposure, risk and environmental vulnerability associated with oil activity [4]. Studies conducted in different international contexts have integrated variables such as well density, sociodemographic indicators, operational status of the infrastructure, geological characteristics, and distances to populated centres or urban infrastructure to estimate levels of exposure and risk [[5], [6], [7]]. These approaches have demonstrated the value of integrating technical, environmental, and social information on geospatial platforms, thereby supporting territorial planning, risk assessment, and environmental decision-making [8].

In the case of Ecuador, the historical presence of wells in urbanised coastal areas, particularly in the province of Santa Elena, necessitates structured, up-to-date, and georeferenced information to enable systematic analysis of the interaction among oil infrastructure, population, and sensitive ecosystems. However, the availability of integrated geospatial datasets that articulate these dimensions remains limited, hindering comparable spatial assessments and the development of support tools for territorial management.

In this context, this study develops and makes available a geospatial dataset that compiles and standardises the technical characteristics of oil wells, along with demographic, environmental, and urban infrastructure indicators, in coastal urban areas. Using geospatial tools and a spatial vulnerability analysis framework, the dataset enables assessment of the proximity between hydrocarbon infrastructure and coastal communities. It is presented as a reusable resource for future research, land-use planning processes, and comparative assessments in territories with similar socio-environmental conditions.

## 3 Data Description

**Fig. 1:**
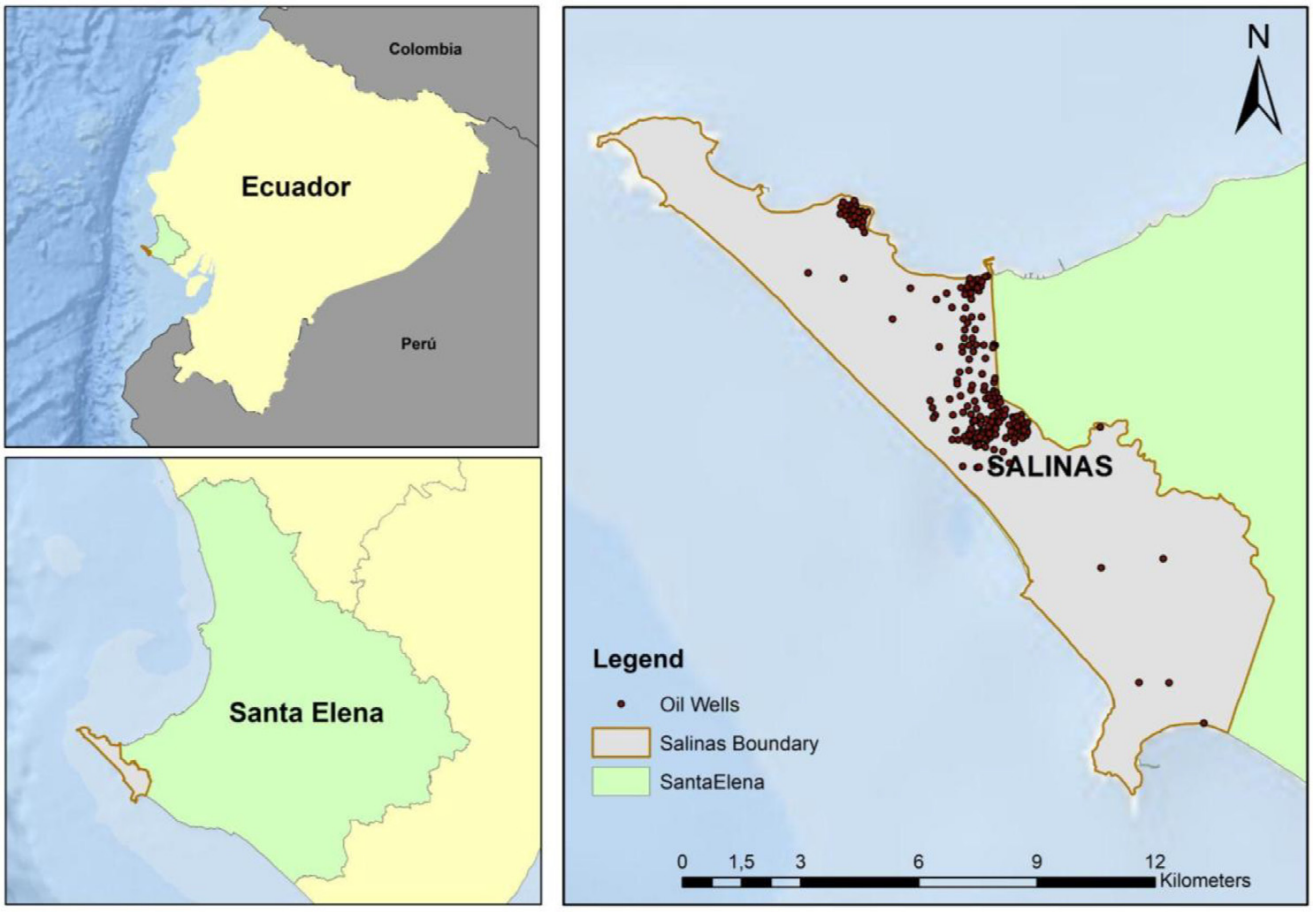
Location of oil wells in the Salinas canton, Santa Elena province, Ecuador.

The dataset comprises the geospatial characterisation of 228 oil wells in the Salinas canton, Santa Elena province, Ecuador. For each well, a set of eleven variables was structured to evaluate its physical, operational, social, and environmental context. The information is presented in geospatial (DatasetOilWell.gpkg) and tabular (Geospatial Dataset Oil Well.csv) formats, compatible with GIS platforms such as QGIS. Fig. 1 shows the spatial distribution of the wells in the coastal urban area.

Table 1 summarises the first records of the dataset, including information on the variables used to assess the environmental and territorial sensitivity of the wells.

**Table 1:** First ten records of the dataset with variables related to environmental sensitivity.

N°	Latitude	Longitude	Well ID	Geological formation	Extraction system	Well status	Safety distance	Oil and multi-product pipelines distance	Gas pipelines distance	Population density	Structures proximity	Sensitive ecosystems	Pipeline interference	VI	E
1	-2.313072222	-80.8822778	ACH001	1	None	2	4	1	1	0	1	0	1	3.5	Low
2	-2.284791667	-80.8834944	ACH002	4	4	3	4	1	1	1	0	1	1	5.2	Medium
3	-2.286783333	-80.8975778	ACH003	1	4	3	4	1	1	1	0	1	1	4.75	Low
4	-2.322363889	-80.874425	ACH004	3	4	3	4	1	1	1	0	1	1	5.35	Medium
5	-2.254747222	-80.8976167	ACH005	None	None	2	4	1	1	1	0	1	1	2.25	Very Low
6	-2.220533333	-80.9556083	ACU001	1	None	2	3	0	0	0	1	1	1	6.75	Medium
7	-2.219266667	-80.9636222	SLS001	2	None	2	4	0	0	1	0	1	1	5.25	Medium
8	-2.220180556	-80.9230083	MEY001	5	None	2	1	0	0	1	1	1	1	6.55	Medium
9	-2.220341667	-80.923575	MEY002	5	1	3	1	0	0	1	1	1	1	8.55	High
10	-2.229888889	-80.9446	ACU002	2	None	2	4	1	1	1	0	1	1	3.25	Low

*VI: Vulnerability Index; E: Equivalence. Lon: Longitude; Lat: Latitude

### 3.1 Variables included in the dataset

**1. Well ID**

Include the registered well identification code.

**2. Geological formation**

The study area is located within the geological structure known as the Santa Elena Uplift, where the Atlanta (AT), Atlanta/Santa Elena (AT/SE), Clay Pebble Beds/Passage Beds/Atlanta (CPB/PB/AT), Passage Beds/Atlanta (PB/AT), Santa Elena (SE), and Santo Tomás (ST) formations predominate [9]. The AT, PB, and ST formations are characterised by interbedded shales, sandstones, and conglomerates, whereas the SE formation is distinguished by siltstone and tuffaceous shale beds. The CPB formation, for its part, is composed of clays, sandstones, limestones, and shales [10]. This latter formation acts as the main reservoir for the Santa Paula and Petropolis oil fields [11]. The specific geological composition of each formation influences the dynamics of hydrocarbon accumulation and extraction, as well as its potential interaction with environmental processes. Each formation was numerically coded (1–6) to integrate it into the vulnerability index, accounting for its lithological and structural differences.

### 3.2 Extraction system

The choice of hydrocarbon extraction system is an important factor in determining operational efficiency and the potential environmental impacts associated with oil production, as these can alter local soils and ecosystems [12]. The dataset recorded the main operating methods used in the study area: Mechanical Pumping (MP), Local Tool (LWT), Plunger Lift (PL), and Swab (SW). Each system was categorised and weighted on a scale of 1 to 4 according to its operational typology.

### 3.3 Well status

This variable classifies the wells in the study area into three production states: Permanently Abandoned (PA), Temporarily Abandoned (TA), and Productive (P). Ninety-eight per cent of the wells correspond to infrastructure associated with the oil field's historical production. However, some of them do not fully comply with local environmental regulations governing permanent or temporary abandonment procedures, as evidenced by inadequate signage or insufficient safety fencing [13]. The assigned scores are 1 for PA, 2 for TA, and 3 for P.

### 3.4 Safety distance

According to the local ordinance, the minimum safety distance around the wellhead or hydrocarbon production facilities must be maintained at a maximum radius of 20 meters [14]. This measure is essential to reduce potential risks and safeguard the population and the surrounding infrastructure. In the dataset, a value of 1 is assigned when the regulations are met and 0 when they are not.

### 3.5 Oil pipeline and multi-product pipeline distance

The study area establishes a buffer zone of 15 meters measured from the centerline of oil and gas pipelines, within which a maximum population density of 120 inhabitants per hectare is permitted. Compliance with this regulation is coded as 1 when the established distance is respected and 0 otherwise.

### 3.6 Distance of gas pipelines

The minimum distance from gas pipelines is established as a risk-prevention measure to prevent potential leaks or gas emissions. This distance is defined as being within a 25-meter radius of the pipeline. In the dataset, a value of 1 is assigned when the distance meets the established criterion and 0 when it does not.

### 3.7 Population density

Population density near oil facilities can increase communities' exposure to potential environmental and health impacts. Several studies have linked proximity to production wells to health problems, including gas emissions, heat stress, vector-borne diseases, dermatological conditions, and cardiovascular or respiratory disorders [15]. A value of 0 is assigned to this variable when population density exceeds the limit permitted by local regulations (120 inhabitants per hectare), and a value of 1 when it remains within the permitted range.

### 3.8 Proximity to infrastructure

This variable includes proximity to educational institutions, health centres, social facilities, road and tourism infrastructure, as well as basic service networks such as drinking water and sewage systems. It is coded as 1 when there is proximity or spatial interaction with this type of infrastructure, and 0 when no interaction is recorded.

### 3.9 Ecosystems and sensitive areas

Within the study area, several ecosystems sensitive to oil activity have been identified, including wetlands, beaches, marine-coastal reserves, oxidation ponds, as well as agricultural, artisanal, and industrial fishing zones, and areas dedicated to salt extraction and production. This variable is represented by a binary code (0–1), where 0 indicates the absence of sensitive ecosystems, and 1 indicates their presence in the immediate vicinity of the well.

### 3.10 Pipeline interference

This variable indicates the presence of structural or maintenance problems in pipelines connected to oil wells across different sectors of the canton. The lack of monitoring and proper maintenance can lead to pipeline ruptures or leaks, posing significant risks to the environment, human health, and the sustainability of local communities. Scores assigned to this variable range from 0 to 1: a score of 0 indicates the presence of structural problems, while a score of 1 indicates no such problems.

## 4 Experimental Design, Materials and Methods

The geospatial dataset of oil wells and socio-environmental vulnerability indicators in coastal urban territories was developed through a structured process in three sequential phases, oriented towards the collection, validation and systematisation of spatial and alphanumeric information.

Phase 1: Collection and selection of variables.

A review and extraction of information was conducted using technical data sheets and official reports on oil wells available from the GAD of the Salinas canton. From these documents, variables related to the geographic location, operational status, and technical characteristics of the wells were identified and systematised, along with relevant social, environmental, and territorial indicators for the analysis of socio-environmental vulnerability in coastal urban environments. Variables were selected based on their relevance to characterising the physical, environmental, and territorial conditions of the study area, as well as their availability and consistency in institutional records.

Phase 2: Georeferencing and field verification

The spatial location of the wells was verified and adjusted using geospatial tools. A Geographic Information System (QGIS) was used to integrate, edit, and cartographically validate the spatial information, and a GPS navigation system was used to verify the provided coordinates and capture geospatial data of the wells' immediate surroundings. Technical field visits were allowed to compare the documented information with current terrain conditions, record updated geographic coordinates, and validate attributes associated with each well, such as its operational status, proximity to infrastructure, and safety conditions. This procedure ensured spatial consistency and greater accuracy in the generated geospatial database.

Phase 3: Index structuring and geodatabase construction.

Following the methodological approach described in [16], the selected variables were normalised to allow comparison on a common scale. Subsequently, relative weights were assigned to each variable based on its relevance to the socio-environmental vulnerability analysis. The weighted sum of the variables yielded a composite vulnerability index, expressed as a percentage for each well analysed. For organisational and reference purposes, the values ​​obtained were classified into four vulnerability ranges: High (≥75%), Medium (50–75%), Low (25–50%), and Very Low (0–25%).

**Fig. 2:**
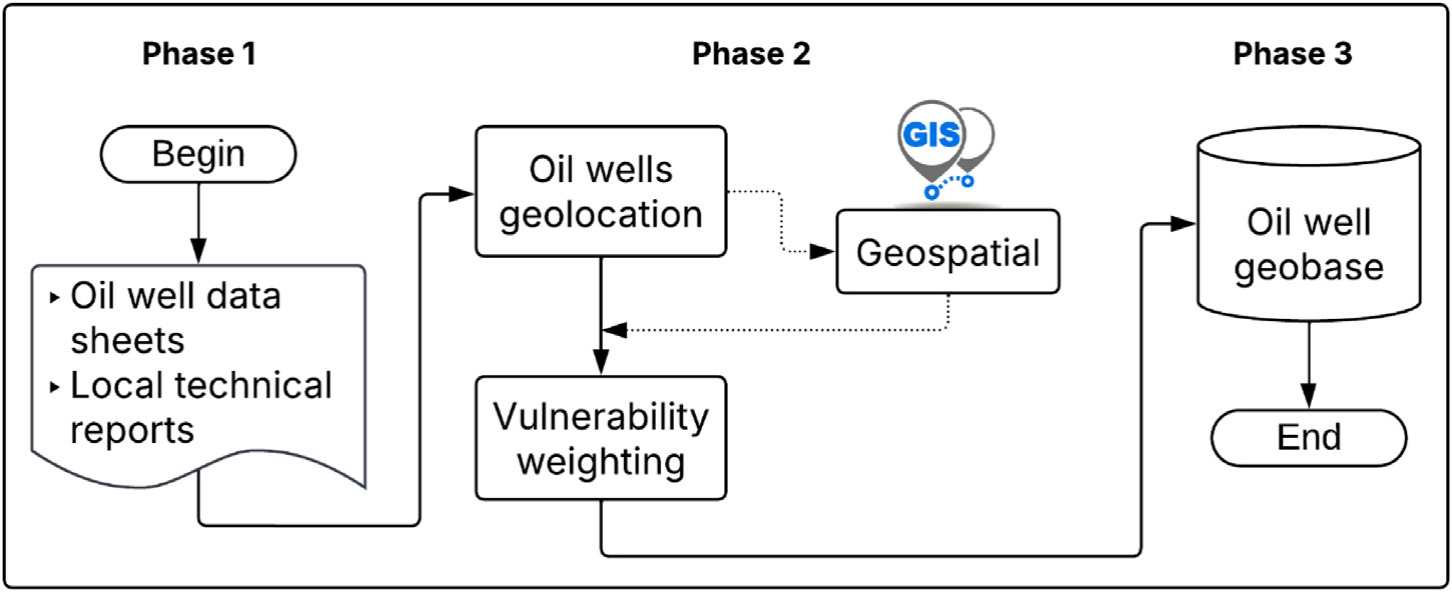
Methodological approach used to obtain and structure the data set.

Finally, all spatial and alphanumeric information was integrated into a structured geodatabase for use in GIS environments, facilitating visualisation, spatial analysis, and the reuse of the dataset in subsequent studies. Fig. 2 presents the methodological scheme used to obtain and organise the dataset.

## Limitations

This dataset addresses the socio-environmental vulnerability associated with the proximity of oil wells in coastal urban areas, using a specific set of variables collected and systematised during the data collection and systematisation process. However, environmental vulnerability is a multidimensional phenomenon that can include additional biological, geomorphological, hydrological, and ecosystemic components, which were not incorporated into this version of the dataset due to limitations in access, availability, and information homogeneity.

Consequently, the scope of the analysis is limited to the indicators collected and processed during the methodological development described. The incorporation of new thematic layers and complementary variables in future updates would broaden the dataset's representativeness and strengthen its potential for comparative studies, more complex spatial simulations, and comprehensive vulnerability assessments in coastal urban contexts.

## Ethics Statement

The authors have reviewed and complied with the ethical requirements for publication in Data in Brief. It is important to note that this study does not involve human subjects, animal experimentation, or data collection from social media platforms.

## CRediT Author Statement

**David Freire:** Conceptualization, Methodology, Investigation, Formal Analysis, Software, Data review, Data curation, Validation, Writing – review & editing. **Paulo Escandón-Panchana:** Conceptualization, Methodology, Investigation, Formal Analysis, Software, Data review, Data curation, Validation, Writing – original draft, Writing – review & editing. **Jorge Magallanes-Tomalá:** Methodology, Investigation Formal Analysis, Software, Validation, Writing – review & editing. **Andrés Velastegui-Montoya:** Conceptualization, Methodology, Investigation, Formal Analysis, Software, Data review, Data curation, Validation, Writing – original draft, Writing – review & editing, Supervision. **Jenny Escandón-Panchana:** Methodology, Investigation, Formal Analysis, Writing – review & editing.

## Data Availability

Mendeley DataGeospatial dataset of oil wells and socio-environmental vulnerability indicators in coastal urban territories (Reference data).
